# 3D-Printed Polymer-Infiltrated Ceramic Network with Biocompatible Adhesive to Potentiate Dental Implant Applications

**DOI:** 10.3390/ma14195513

**Published:** 2021-09-23

**Authors:** Ľudmila Hodásová, Carlos Alemán, Luís J. del Valle, Luis Llanes, Gemma Fargas, Elaine Armelin

**Affiliations:** 1 Departament d’Enginyeria Química, IMEM Group, Campus Diagonal Besòs (EEBE), Universitat Politècnica de Catalunya, C/ Eduard Maristany, 10-14, Building I, 2nd Floor, 08019 Barcelona, Spain; ludmila.hodasova1@upc.edu (Ľ.H.); carlos.aleman@upc.edu (C.A.); luis.javier.del.valle@upc.edu (L.J.d.V.); 2 Departament de Ciència i Enginyeria de Materials, CIEFMA Group, Campus Diagonal Besòs (EEBE), Universitat Politècnica de Catalunya, C/ Eduard Maristany, 10-14, Building I, 1st Floor, 08019 Barcelona, Spain; luis.miguel.llanes@upc.edu; 3 Barcelona Research Center for Multiscale Science and Engineering, Campus Diagonal Besòs (EEBE), Universitat Politècnica de Catalunya, C/ Eduard Maristany, 10-14, Building I, Basement Floor, 08019 Barcelona, Spain

**Keywords:** robocasting, yttria-stabilized zirconia, acrylate polymers, X-ray microtomography, MG-63 human cell

## Abstract

The aim of this work was to prepare and characterize polymer–ceramic composite material for dental applications, which must resist fracture and wear under extreme forces. It must also be compatible with the hostile environment of the oral cavity. The most common restorative and biocompatible copolymer, 2,2-bis(p-(2′-2-hydroxy-3′-methacryloxypropoxy)phenyl)propane and triethyleneglycol dimethacrylate, was combined with 3D-printed yttria-stabilized tetragonal zirconia scaffolds with a 50% infill. The proper scaffold deposition and morphology of samples with 50% zirconia infill were studied by means of X-ray computed microtomography and scanning electron microscopy. Samples that were infiltrated with copolymer were observed under compression stress, and the structure’s failure was recorded using an Infrared Vic 2D^TM^ camera, in comparison with empty scaffolds. The biocompatibility of the composite material was ascertained with an MG-63 cell viability assay. The microtomography proves the homogeneous distribution of pores throughout the whole sample, whereas the presence of the biocompatible copolymer among the ceramic filaments, referred to as a polymer-infiltrated ceramic network (PICN), results in a safety “damper”, preventing crack propagation and securing the desired material flexibility, as observed by an infrared camera in real time. The study represents a challenge for future dental implant applications, demonstrating that it is possible to combine the fast robocasting of ceramic paste and covalent bonding of polymer adhesive for hybrid material stabilization.

## 1. Introduction

Nowadays, the most important metal used in dental implants and jaw fixation is titanium [1]. Per-Ingvar Brånemark, a Sweden physician and research professor, successfully integrated a titanium screw in a human subject in 1965, changing the dental landscape [2]. Since then, several materials have been investigated and not only pure metals were explored but also metals, ceramics modified with biocompatible coatings, and polymers. Some examples include calcium phosphate and hydroxyapatite (HA) [3,4,5,6]; some metal alloys, such as nickel-titanium (NiTi), stainless steel, and Vitallium (a cobalt-chrome alloy composed of 65% Co, 30% Cr, and 5% Mo) [7]; ceramic cements like alumina (Al_2_O_3_), spinel (MgAl_2_O_4_) and zirconia (ZrO_2_) [4]; and polymers such as sulfonated poly(ether-ether-ketone) (PEEK), poly(methylmethacrylate) (PMMA), and polylactic acid (PLA) [8,9,10,11].

Zirconia (ZrO_2_) was first reported as being used in dental implantation surgery in 1975 when Cranin and co-workers employed zirconia and alumina (Al_2_O_3_) to coat Vitallium alloy [7]. Many decades later, several studies revealed some important advantages of zirconia with respect to other materials, for example, minimal local or systemic adverse reactions, biocompatibility with bone and soft tissues, excellent tissue response, and superior esthetic appearance, among others [12,13,14,15]. Zirconia has excellent mechanical and esthetic properties for orthodontic restorations, such as tooth-like natural coloration and it is suitable for the fabrication of single crowns, fixed partial dentures, and implant abutments in the dentistry field. However, the two most important drawbacks involved with this type of ceramic, from a clinical point of view, are chipping fractures and spontaneous failure caused by accelerated aging (known as low-temperature degradation) [16,17,18,19,20].

From ancestral civilizations to the present day [1], there has been a continuous search for the best biomaterial for dental implants and orthodontic tools for oral cavity and jaw fixation. For example, Schünemann et al. [15] have recently discussed the relevance of the correct ceramic surface modification to enhance osseointegration properties. Satisfactory bone-to-implant contact (BIC) values and a substantial reduction in bacterial biofilm adhesion were found in machined zirconia implants when compared to titanium ones. Moreover, it was reported that zirconia-based implants with bioactive coatings, such as calcium phosphate ceramics and bioactive glasses, can speed up the osseointegration process for both zirconia and titanium implants.

Currently, zirconia restorations are manufactured by either soft- or hard-milling processes. However, nowadays, additive manufacturing is at the top of the most important fabrication methods for the fast production and reproducibility of pieces [21,22,23]. 3D-printing technology permits facile assembly and, depending on the scaffold configuration, it can also be used to control the mechanical stability of the pieces by combining different materials (ceramic–polymer, ceramic–metal, metal–polymer) and different geometries [24,25,26].

While advancements in 3D-printing technology have increased the effectiveness of dental prototype fabrication, the desired final property of the material itself is still the key aspect setting the limitations of the fabrication process. Fortunately, the authors of this study were recently able to create—following a robocasting route—a new polymer-infiltrated ceramic network (PICN) with 50% infill of solid yttria-stabilized tetragonal zirconia (3Y-TZP) filaments and 50% of macropores filled with methacrylate polymer, in a cubic geometry [27]. The work demonstrated that different architectures can be explored, combining the robocasting process and the direct interfacing of biocompatible polymer adhesives inside ceramic dental implants. This approach should allow the reduction in Young’s modulus of zirconia (~200 GPa) to values closer to dentin (20–25 GPa) [28,29,30], without the loss of adaptive modulation to teeth [31]. Therefore, the aim of the present work is to investigate the morphology, crystalline structure changes, and hardness of PICN after 3D-printing fabrication, as well as the biocompatibility of the system when in contact with human cells.

## 2. Materials and Methods

### 2.1. Materials

Zirconia powder was provided by SEPR Saint-Gobain ZirPro under the commercial name CY3Z-R, (Le Pontet Cedex, France). It consists of 3 mol% yttria-stabilized tetragonal zirconia polycrystal (hereafter denoted as 3Y-TZP), with a particle size of about 300 nm. The ceramic density is 6.05 g·cm^−3^, after the sintering process. Pluronic^®^ F-127 hydrogel (25% *w*/*v*); γ-MPS (3-(trimethoxysilyl)propyl methacrylate); Bis-GMA (bisphenol A glycerolate dimethacrylate); TEGDMA (triethylene glycol dimethacrylate) and BPO (benzoyl peroxide, Luperox^®^A75) were all supplied by Sigma-Aldrich (Madrid, Spain) and were used to prepare the polymer-modified pieces.

### 2.2. Preparation of 3D-Printed Cubic Samples, Ceramic Functionalization, and Copolymer Covalent Deposition

The detailed procedure for scaffold fabrication was as recently reported elsewhere [27]. In brief, 3Y-TZP powder (70 wt. %) was mixed with Pluronic^®^ F-127 hydrogel (30 wt. %) using a D-VM 16 vacuum mixer from Harnisch-Rieth, (Winterbach, Germany). Aiming to prevent drying, immediately after mixing, an extrusion syringe was filled with the paste, then the paste was extruded by a 3D printer using a 3D Dima Elite dispenser (Nordson Dima, Deurne, Netherlands), with a cylindrical nozzle of 800 µm and equipped with DimaSoft CAD/CAM software. Extrusion was followed by a 5-day drying period before sintering. After a two-step sintering process (at 700 °C for 1 h and at 1450 °C for 2 h), zirconia cubes with dimensions of 1.2 × 1.2 × 0.8 cm^3^ and with 50% infill of zirconia filaments were obtained. Afterward, the 3D-printed scaffolds were submerged in a previously stirred solution of γ-MPS (24 mmol of liquid silane in 100 mL of 3:1 ethanol:water volume ratio) for 1 h, then they were removed and left to partially dry before the acrylate polymerization. Then, the silane functionalized pieces were immersed in a viscous solution with 39.5 wt. % of Bis-GMA, 59.5 wt. % of TEGDMA monomers and 1 wt. % of BPO initiator (previously stirred for 1 h at room temperature); and left to penetrate the scaffold for an additional 1 h. The hybrid system was then removed from the solution and placed in an aluminum foil-covered glass petri dish to complete the curing process at 115 °C for 10 h in a vacuum oven. The hybrid material thus produced received the name PICN, as previously indicated.

### 2.3. Scaffold Characterization

The crystalline structure of 3Y-TZP was investigated by means of X-ray diffraction (XRD). The equipment used was a Bruker D8-Discover diffractometer (Billerica, MA, USA) with a vertical goniometer (Bragg-Brentano configuration θ-2θ), XYZ-motorized stage mounted on a sample holder, and provided with a PSD Lynx-Eye detector. The data were collected in the 2θ range from 10° to 80° with an angular step of 0.02° at 1 s per step and a fixed incidence angle of 1°. Cu Kα radiation was obtained from a Cu X-ray tube operated at 40 kV and 40 mA. The average crystal size was estimated using the Debye-Scherrer equation:

t = Kλ/B cos θ_B_ (1)

where t is the size of the crystalline domains, K is a dimensionless shape factor (default value = 0.9 when using a Cu Kα source), λ is the X-ray wavelength (0.15417 nm), B is the line broadening at half of the maximum intensity, and θ_B_ is the Bragg angle for tetragonal and monoclinic diffraction peaks.

The topography of zirconia filaments and grain size were investigated using scanning electron microscopy (SEM). The study was carried out using a focused-ion-beam Zeiss Neon 40 scanning electron microscope (Carl Zeiss, Oberkochen, Germany) and equipped with an energy-dispersive X-ray (EDX) spectroscopy system. Secondary electrons detector (SE) was employed to study the topography of the surfaces and an immersion lens detector (InLens) was preferred for high lateral resolution. The scanning was carried out at 5 kV. The samples were mounted on a double-sided adhesive carbon disc and sputter-coated with a thin layer of carbon to prevent sample charging problems.

The structure, pore distribution, and overall architecture of zirconia 3D-printed scaffolds (50% infill) were analyzed with an X-ray computed microtomography (micro-CT) Skyscan 1272 by Bruker (Kontich, Belgium). The measurement was performed at a source voltage of 100 keV and a current of 100 µA, with an isotropic pixel size of 4.6 µm. The analysis and the scan stacking were performed with Nrecon and CTAn software from Bruker.

The Vickers hardness of the empty 3Y-TZP scaffold and the copolymer-infiltrated composite were obtained using a DuraScan 10 G5 unit by Emco-Test (Kuchl, Austria). Abraded 3D-printed samples were used, with and without infiltrated copolymer. The hardness imprint was made on the filament area of each sample using an applied load of 7 kN; thus, the data measured are reported as HV10.

The failure mode of the 50% infill 3D scaffold, 100% infill 3D scaffold, and polymer infiltrated 3D scaffold under pressure was observed with an Infrared Vic 2D^TM^ camera and analyzed with Vic 2D software from Correlated Solutions, Inc. (Irmo, SC, USA). Vic-2D software uses optimized algorithms to provide full-field displacement and strain data for mechanical testing on planar samples. Definite in-plane movement can be determined for every point within the measurement surface, as well as by using the Lagrangian strain tensor. For this analysis, the samples were abraded to attain straight sides that were exposed to the camera recording. Compression tests were conducted using an Instron 8511 universal testing machine, under a displacement rate of 0.1 mm/s. As a result, the high-stress areas and crack propagation were recorded in real time until the sample cracked or until a maximum applied force of 7 kN was reached.

The chemical composition of the adhesive was investigated by infrared spectroscopy. The infrared spectra of Bis-GMA/TEGDMA copolymer and pure monomers were measured with an FTIR 4700 Jasco spectrophotometer (Madrid, Spain). The equipment is coupled to an attenuated total reflection (ATR) accessory (Specac model MKII Golden Gate Heated Single Reflection Diamond ATR). For this, 32 scan accumulations and a resolution of 8 cm^−1^ were chosen for spectra acquisition in the wavenumber range from 4000 to 600 cm^−1^. The spectra are reported as a percentage of transmittance versus wavenumber.

### 2.4. Human Cells Adhesion and Proliferation

MG-63 cells (derived from human osteosarcoma; ATCC) were cultured in Dulbecco’s modified Eagle’s medium (DMEM). It contains 4500 mg/L of glucose, 110 mg/L of sodium pyruvate, and 2 mM of L-glutamine, complemented with 10% fetal bovine serum (FBS); 50 U/cm^3^ penicillin, 50 mg/mL streptomycin, and L-glutamine 2 mM at 37 °C, in a 10% humidified atmosphere with CO_2_ and air in the proportion of 5% and 95%, respectively. Culture media were changed every two days, as standard protocol. For the sub-culture, cell monolayers were rinsed with PBS solution (phosphate buffer saline) and detached by incubating them with 2 mL TrypLE^TM^ (Gibco, Amarillo, TX, USA) for 2–5 min at 37 °C. The incubation was stopped by re-suspending in 5 mL of fresh medium. For cell counting, a Neubauer camera and a dye (trypan blue, 4%) were employed.

3D scaffolds were placed in 12-well tissue culture plates and sterilized by exposure to UV light for 15 min for each side of the cube. A culture medium (4 mL) was added to each well to cover the scaffolds. Then, 100 µL containing 2 × 10^4^ cells/well to rate cell adhesion, and 5 × 10^4^ cells/well for a cell proliferation assay, were seeded in each well. The plates were incubated for 24 h to check the cellular adhesion and, further, for 7 days to determine the cell proliferation. In parallel, the control was performed in each without any scaffold.

The percentage of cells adhered (after 24 h) and proliferated (after 7 days) was determined through an MTT (3-(4,5-dimethylthiazol-2-yl)-2,5-diphenyltetrazolium bromide) assay [32]. The procedure consisted of washing each well per triplicate with PBS and adding 4 mL of culture medium to each well. Afterward, 200 µL of MTT (3 mg/mL) were also added and incubated for 4 h. Then, the samples were washed again (twice) with PBS and the specimens were deposited in a new plate. Finally, 2 mL of dimethyl sulfoxide (DMSO) were subsequently added to each well to measure the absorbance (570 nm) in a microplate reader (Biochrom EZ-Read 400, Fisher Scientific, Madrid, Spain), after 15 min of gentle stirring. Three replicates were evaluated, and the corresponding values were averaged for its plot representation; statistical analysis was performed by ANOVA software, followed by the Tukey test (OriginPro v8).

## 3. Results and Discussion

### 3.1. Hybrid Material Characterization

**Figure 1:**
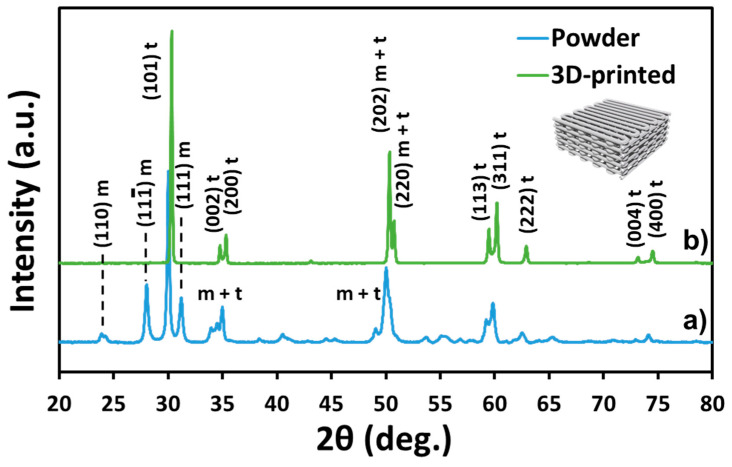
XRD spectra with an incident angle of 1° of: (**a**) 3Y-TZP powder and (**b**) 3D-printed sample, after the robocasting and sintering process, up to 700 °C and 1450 °C. The inset represents the cubic geometry designed by layer-by-layer zigzag filament deposition to create macropores for further copolymer infiltration.

The fabrication of dense and geometrically complex ceramic structures through 3D-printing technology by using polymer-derived ceramic precursors is relatively easy with 100% infill of ceramic deposition. However, the preparation of 3D-printed highly porous structures is rather difficult, due to the sample deformation. Considering that the objective of this study was the development of a hybrid material intended for dental applications, the most important properties to be evaluated for the new PICN material are the mechanical resistance and osseointegration via a cell viability test. The first property will be affected by the piece’s geometry, the proper infiltration of polymer adhesive to create the PICN structure, and the crystal structure of the zirconia filaments after the robocasting and sintering processes. In this regard, the geometry was fixed to have the maximum percentage of macropores as possible without the loss of the CAD/CAM 3D cubic design, characterized by a layer-by-layer zigzag filament deposition and the number of layers being 10 (Figure 1, inset image). Proper polymer infiltration has been discussed in depth in our previous work [25].

It is well known that the monoclinic and cubic phases of zirconia are less mechanically stable than the tetragonal one, and that phase changes are usually observed after aging, hydration, or temperature increase [33,34,35]. The XRD spectrum of the 3D-printed material shows typical changes that have been reported in the literature [25]. After the robocasting and sintering processes, both tetragonal and monoclinic phases coexist. However, in this study, the comparison of spectra from the powder and the 3D-printed piece (Figure 1) indicates that the tetragonal phase is predominant, whereas the monoclinic one decreases substantially [34]. Moreover, a lack of broadening of the (111) peak confirms that there is no overlapping with other phases (e.g., the rhombohedral one). Similarly, the (002) and (200) peaks at 34.8° and 35.3°, associated with the tetragonal phase, have converged into pure tetragonal phases when compared to the spectrum of the powder. This further indicates that this phase is concentrated in regions of less than 1 µm from the surface; it should be noted that, for an incidence angle of 1° with Cu Ka radiation, the penetration depth is ~0.3 µm. The XRD peaks of the sintered 3D printed body were indexed against the standard reflection pattern of tetragonal (t-ZrO_2_; JCPDS #50-1089) and monoclinic (m-ZrO_2_; JCPDS #37-1484) zirconia.

The crystalline size of the tetragonal lattice was found to be 35 nm and 51 nm, before and after robocasting, respectively (Table 1). From these data, it is clear that the tetragonal structure is formed after the rearrangement of monoclinic nanocrystallites. The size of these nanocrystals tends to be slightly higher in the case of the 3D-printed samples than in the case of the powder ones.

**Figure 2:**
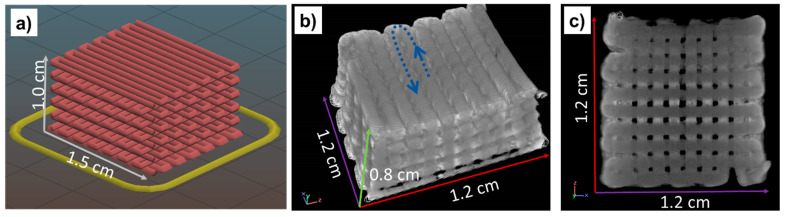
(**a**) CAD/CAM 3D design of the simple cubic geometry of zirconia. Reprinted from Ref. [27] with permission; Copyright Elsevier 2021, (**b**) three-dimensional micro-CT image and (**c**) cross-sectional micro-CT image of the sintered 3D-printed scaffold, with a 50% feed infill of zirconia. The dashed arrow in (**b**) indicates the direction of filament deposition in one single layer.

The computed tomography technique (commonly referred to as micro-CT) allowed 3D- and 2D-investigations of the marginal and internal gaps produced by the computer-aided manufacturing of zirconia scaffolds, within the range of a few micrometers (Figure 2). The ceramic scaffold obtained was found to be very stable, even though 50% of the volume of the cubic pieces—generated through robocasting of ten zig-zag layers of zirconia filaments (Figure 2a)—is composed of voids. In Figure 2b, it is possible to envisage the direction of the filament deposition. The images also reveal the shrinkage of the whole structure after the sintering step, corresponding to a contraction of about 20%. According to the micro-CT image of Figure 2c, the average size of macropores is 380.11 ± 51.77 µm.

**Figure 3:**
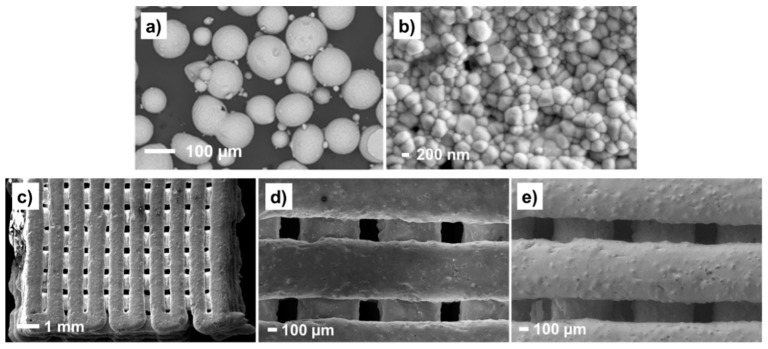
SEM micrographs of (**a**) 3Y-TZP powder; (**b**) zirconia paste after robocasting and sintering; (**c**) 3D-printed zirconia filaments and macropores created by the robocasting architecture; (**d**,**e**) high magnification micrographs with a detail of 3 well-arranged filaments. All images were taken with an InLens detector, with the exception of Figure (**e**) where the secondary electrons detector (SE) was employed to better visualize the surface texture of the filaments.

The successful deposition of zirconia filaments was only possible thanks to the mixing of 3Y-TZP powder with Pluronic^®^ hydrogel under vacuum, which helped to avoid bubbles, and, thanks to the employment of an adequate printer nozzle (800 µm in diameter), which avoided particle agglomerations and the formation of defects during the printing process. In Figure 3, the morphology of the 3Y-TZP zirconia powder has been compared with that obtained after the robocasting process. As can be seen, the zirconia powder has a spherical morphology with particle diameters at a micrometric scale (109 ± 25 µm, Figure 3a). After the robocasting deposition and sintering, the particles have been reduced to 448 ± 71 nm (Figure 3b). In the same micrograph, inter-particle voids left by the hydrogel degradation can be also visualized. The porosity of such dense filaments was determined by gas displacement pycnometry in a previous work by the authors, being very low (3%) [27]. Thus, such microporosity is not presumed to affect the copolymer infiltration of macropores, strategically created to reinforce the 3Y-TZP zirconia scaffolds.

Once the material was printed and sintered at high temperatures, the SEM images at low magnification revealed the good stability of the zirconia filaments (Figure 3c), corroborating that observed previously by micro-CT (Figure 2b). The surface topography was inspected by employing the InLens detector (Figure 3d) and secondary electrons detector (Figure 3e). As can be seen, the filaments exhibit a granular texture and are perfectly deposited layer-by-layer.

**Figure 4:**
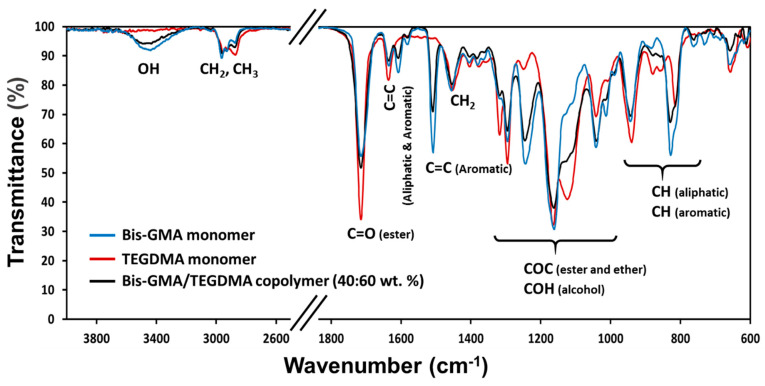
FTIR spectra of Bis-GMA/TEGDMA copolymer in the PICN sample, after curing at 110 °C, and the respective monomers used for the copolymer synthesis.

The PICN pieces, with the zirconia scaffold voids filled by the copolymer adhesive, were polished and the infiltrated polymer was analyzed by ATR-FTIR spectroscopy, comparing the main chemical bonds of cured copolymer with the pristine monomers (Figure 4); the O–H stretching appearing at ~3400 cm^−1^ belongs to the Bis-GMA units. This compound also produces O–H bending vibrations but the latter couple with other vibrations and produce complex bands in the fingerprint region, coinciding with that of ester and ether groups (1000–1200 cm^−1^). The spectra of monomers also show a sharp absorption band, corresponding to the C=C stretching wavenumber at 1635 cm^−1^, which has been reduced in the copolymer spectrum, evidencing successful radical copolymerization. The presence of aromatic rings from the Bis-GMA monomer also manifests at the region between 900 and 800 cm^−1^. Moreover, the extensive presence of methylene groups can be confirmed by the absorption band at 1452 cm^−1^, corresponding to scissoring frequencies [36,37]. The intensity of functional groups associated with the Bis-GMA and TEGDMA units depends on the copolymer ratio.

### 3.2. Mechanical Tests

The authors of this study have previously demonstrated that 3D-printed zirconia with 50% zirconia infill and PICN (3D-printed zirconia 50% infill, reinforced with Bis-GMA/TEGDMA copolymer) exhibit different mechanical resistance to compression forces [27]. In this work, the reasons for such distinct behavior have been elucidated by recording the deformation of filaments in real time during compression tests, using an infrared camera.

**Figure 5:**
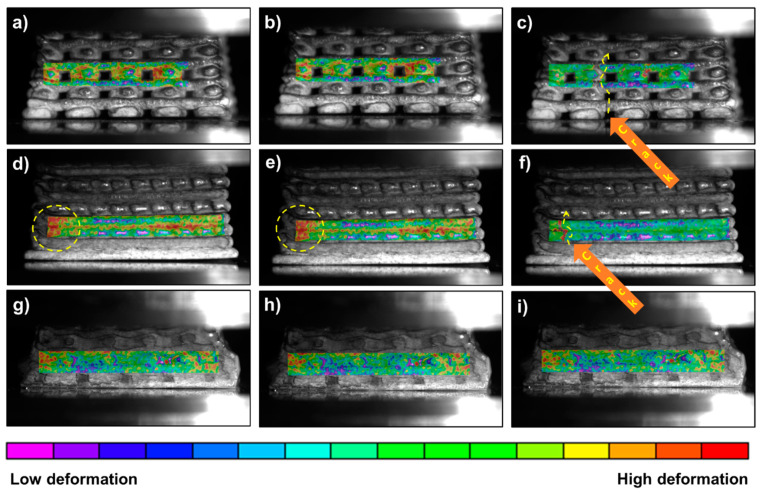
Digital images progress of deformation in the 3D-printed scaffolds, under a compression experiment: (**a**–**c**) 50% infill of 3D-printed zirconia, (**d**–**f**) 100% infill, (**g**–**i**) PICN sample (50% infill of 3D-printed zirconia with infiltrated copolymer). Dashed yellow arrows indicate the direction of filaments breaking, orange arrows indicate the crack propagation and yellow dashed circles show the zone with high deformation under compression, characterized by an deep red color.

The new material is intended to be used in the dentistry field, combining biocompatible ceramic and polymer adhesive. The dental implants are subjected to direct compression and abrasion forces with teeth. Therefore, it is important to evaluate its hardness and mechanical response under compression forces. Vickers hardness tests were conducted on the 50% infill scaffold and the polymer-infiltrated one. The Vickers hardness of the hollow scaffolds (50% infill) was 1414 ± 176 HV10 whereas, for the PICN scaffolds, it was lower (1078 ± 49 HV10). The lower hardness of the PICN sample can be explained by its higher flexibility and resistance to breaking under pressure, due to the filling of the zirconia macropores with Bis-GMA/TEGDMA copolymer, which is a softer material. In summary, the filled macropores transform a rigid and brittle ceramic into a more compliant and soft material. Lin *et. al*. [38] have reported Vickers hardness values of 14.5–24.6 HV, for different compositions of Bis-GMA, TEGDMA, and urethane dimethacrylate 3D-printed 100% infill samples. Other authors have reported different grades of “dense” polymer-infiltrated ceramics varying from 59 to 72% [39]. The hardness (HV5) decreased by a factor of 3.1 GPa to 6.1 GPa for 72% infill and 59% infill, respectively, compared to the 100% dense ceramic (6.41 GPa). Obviously, the absolute values cannot be compared to those obtained in the present work, due to different architectures and polymer-ceramic compositions. However, the tendency is the reduction of hardness in all cases. Thus, it is expected that the copolymer filling can absorb part of the load applied to the rigid zirconia filaments, reducing the intrinsic hardness of the ceramic. The hypothesis of the copolymer acting as a “safety cushion”, preventing crack propagation and securing the desired material flexibility, was supported by the response recorded using an infrared camera (Figure 5 and Videos S1–S3 in the Supplementary Information). Here, three types of 3D-printed samples were evaluated: 50% zirconia infill (Figure 5a–c, Video S1); 100% zirconia infill (Figure 5d–f, Video S2); and PICN (Figure 5g–i, Video S3).

The breaking of the first filament and crack propagation starts at 0.55 kN in the case of zirconia scaffolds with macropores (Video S1). Failure initiates in a filament at the bottom of the cubic piece and extends to the top face very quickly (Figure 5c). As expected, the 100% infill 3D-printed sample was found to be more resistant than the 50% infill one. In this case, the deformation is distributed along filaments placed in the middle of the sample, being one of the edges, indicated by the red color and highlighted by circles in Figure 5d,e, the zone subjected to higher stresses. The cracking and, soon afterward, the complete breaking of the samples with 50% and 100% infill is indicated with an orange arrow in Figure 5c,f. For the latter percentage, complete breaking occurs under 1.4 kN of compression force.

As can be seen in Figure 5g–i, for the same test with the PICN sample, the copolymer holds up the filaments and reduces the amount of stress applied to the sample. With the images and the video recorded (Video S3) for the piece cross-section, it is possible to visualize that the blue zones (low deformation) are located in the interface of the zirconia filaments and polymer adhesive. Such observations of the compression-induced cracks indicate that the polymer network causes lower crack deflection than the 100% infill ceramic material (Video S2). Moreover, as reported previously [27], the 3D-printed sample with infiltrated polymer achieves values of maximum strength that are twice those observed for pieces without polymer (Table 2), without breaking (i.e., it resists compression forces higher than 1 kN). Altogether, these findings led to the conclusion that a PICN hybrid material can be a good candidate to replace pure zirconia implants in the near future. For the dynamic compression deformations in real time, three videos have been included below, corresponding to the 50% infill, 100% infill, and PICN scaffolds. Table 2 summarizes the stress–strain mechanical properties until complete breakdown.

### 3.3. In Vitro Human Cell Adhesion and Proliferation

**Figure 6:**
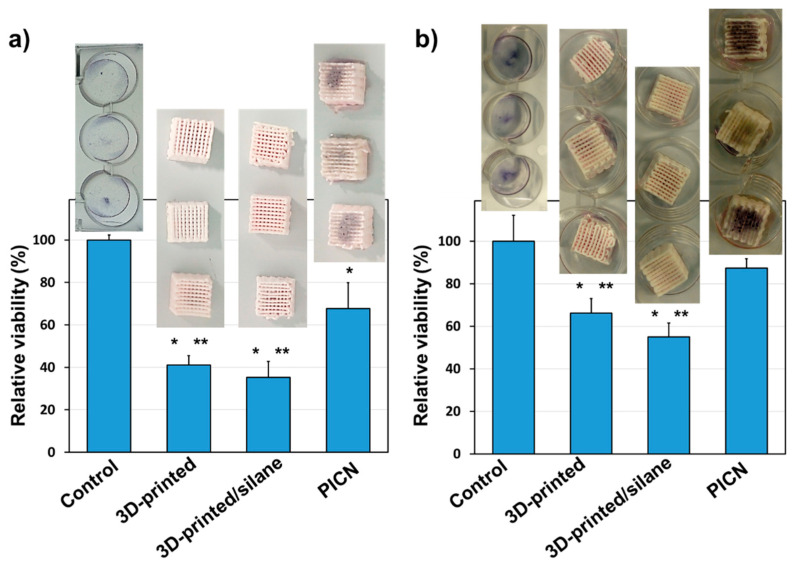
Viability of osteogenic MG-63 cells in the 3D scaffolds. Quantification of cellular adhesion (**a**) and proliferation (**b**). Above each bar, images of the MTT reaction by viable cells, appearing as diffuse or dark spots, are shown. * *p* < 0.05 vs. control, ** *p* < 0.05 vs. copolymer (ANOVA–Tukey’s test).

In a previous study by the authors, it was shown that *Escherichia coli* and *Streptococcus salivarius* bacteria are not able to proliferate on the 3D-printed PICN scaffolds with Bis-GMA/TEGDMA acrylate adhesive [27]. This is an important issue since bacterial infections can cause the rejection of dental implants due to their spread to the jawbone and other structures of the oral cavity, causing a risk of peri-implantitis. In this study, the cell viability of the new system to support the bone and surrounding tissue integration of the new ceramic–organic material was explored. Figure 6 shows the percentages of viability for adhesion and proliferation of the bone-derived epithelial cells (MG-63 cells) that were seeded on the 3D scaffolds. Low adhesion percentages of 41 ± 5% and 35 ± 7%, respectively, are shown in zirconia scaffolds (3Y-TZP) and in zirconia scaffolds with a silane coating (γ-MPS) (Figure 6a). These percentages were significantly lower (*p* < 0.05) than those measured for cell adhesion in the zirconium scaffolds with a coating of silane and copolymer (Bis-GMA/TEGDMA), i.e., 68 ± 12%. Although, this percentage is high, a significant part of the cells seeded in the well-drained area toward the plate surface, suggesting that a block with a larger surface area on the upper face could improve the percentage of adhesion and retention of the cells in the 3D scaffold. The proliferation results indicate that the three types of 3D scaffolds prepared were compatible with cell growth (Figure 6b). The percentages of cell viability at 7 days of culture were 66 ± 7% and 55 ± 7%, for zirconia scaffolds (3Y-TZP) and zirconia scaffolds with a silane coating (γ-MPS). However, they were significantly lower than the control (*p* < 0.05). In this same direction, cell proliferation is noticeable in the 3D scaffolds coated with silane and copolymer, where the percentage of viability was 87 ± 5%, which was not different from the control. Qualitative support of the above findings and statement is given in Figure 6. It shows digital photographs of the 3D scaffolds with viable cells visualized as dots or diffused dark coloration on the surface of the scaffolds, this being quite pronounced in the zirconia scaffold with a silane coating and Bis-GMA/TEGDMA copolymer. It can be concluded that the best results, regarding both adhesion and cell proliferation, were obtained for PICN samples.

Several works have demonstrated the viability of zirconia material towards human-osteoblast cells [14,39,40]. Carinci et al. [41] were able to express various genes from an osteoblast-like cell line (MG-63) cultured on zirconium oxide discs, proving that the ceramic surface is able to promote important cell functions, such as immunity, vesicular transport, and cell cycle regulation. On the contrary, silane compounds do not significantly affect cellular adhesion and viability, they are preferably used as a coupling agent for active biomolecules, like the polysaccharides and proteins used as coatings for implants [42,43].

In a very recent study, reported by ours [44], a catechol-ethylene glycol dimethacrylate copolymer adhered to Y-TZP zirconia discs was tested with MG63 cells, finding relative viability of 100% and 93% for cell adhesion (24 h) and cell proliferation (7 days), respectively. In the present study, the relative viability of MG63 cells was 87 ± 5% (Figure 6b), thus validating the prompt biocompatibility of the acrylate copolymer.

Therefore, the insertion of acrylate copolymers on zirconia has demonstrable advantages for bioactivity, since they have the ability to induce the cell adhesion that is essential for subsequent bone proliferation. The PICN samples studied also display a good correlation regarding structural integrity and biocompatibility. Future work needs to be performed in order to focus on its application in clinical practice. In this sense, mechanical properties, together with cell viability and reduced bacteria proliferation as published elsewhere [27], should be proved in complex 3D-printed geometry that is used for dental implants.

## 4. Conclusions

Here, the successful obtaining of a stable and mechanically resistant PICN 3D-printed scaffold with a biocompatible copolymer acrylate inside the structure macropores was described. The superior cohesion of 3Y-TZP particles in the ceramic layer-by-layer filaments, after sintering, together with the very stable zigzag ceramic configuration, strategically designed to create the 50% infill cubic structures, led to the obtaining of promising hybrid material for dental applications.

For the first time, it was possible to demonstrate that the copolymer infiltrated among the ceramic filaments, created by CAD/CAM design, acts as a mechanical stabilizer and adhesion promoter at the same time.

The compression-testing, combined with the in situ observation of cracking phenomena with an infrared camera, led to the conclusion that PICN samples have a larger capability to resist higher deformation than 50% infill ceramic scaffolds. Moreover, the presence of the polymer also aids in decreasing the ceramic hardness, both properties being desirable for the use of zirconia material in the dentistry field.

The results of MG-63 cell adhesion and proliferation of PICN, compared to 3D-printed zirconia without copolymer adhesive, are also a positive point for future biomedical applications.

## Figures and Tables

**Table 1:** Application of the Debey–Scherrer equation ^1^ for 3Y-TZP powder and 3D-printed zirconia cubes.

Sample	Phase	2θ	Cos (θ)	B (rad)	t (nm)
3Y-TZP powder	Monoclinic	28	0.970	0.005	28
	Tetragonal	30	0.966	0.004	35
3D-printed	Monoclinic	-	-	-	-
	Tetragonal	30	0.966	0.003	51

^1^ Data collected from Equation (1).

**Table 2:** Stress–strain data from the compression test ^1^.

Sample	σ_max_ (MPa)	σ_break_ (MPa)	ε_compression_ ^2^ (%)
3D-printed zirconia 50% infill	86	51	4.1
3D-printed zirconia 100% infill	124	124	5.2
PICN	102	102	5.2

Notes: ^1^ σ = strength, ε = percentage of compression resistance until breaking or with a maximum of compression force. ^2^ Maximum compression percentage until the sample’s breaking.

## Data Availability

Upon request to the corresponding authors.

## Supplementary Materials

The following are available online at https://www.mdpi.com/article/10.3390/ma14195513/s1, Video S1: 50% infill.mp4, Video S2: 100% infill.mp4, Video S3: PICN scaffold.mp4.
